# Interpretable machine learning model for early morbidity risk prediction in patients with sepsis-induced coagulopathy: a multi-center study

**DOI:** 10.3389/fimmu.2025.1552265

**Published:** 2025-03-03

**Authors:** Ruimin Tan, Chen Ge, Jingmei Wang, Zinan Yang, He Guo, Yating Yan, Quansheng Du

**Affiliations:** ^1^ School of Clinical Medical, North China University of Science and Technology, Tangshan, Hebei, China; ^2^ Critical Care Department, Hebei General Hospital, Shijiazhuang, Hebei, China; ^3^ Critical Care Department, Handan Central Hospital, Handan, Hebei, China; ^4^ School of Graduate, Hebei Medical University, Changan, Shijiazhuang, Hebei, China

**Keywords:** machine learning, sepsis-induced coagulopathy, pathogenic factors, predictive model, multi-center study

## Abstract

**Background:**

Sepsis-induced coagulopathy (SIC) is a complex condition characterized by systemic inflammation and coagulopathy. This study aimed to develop and validate a machine learning (ML) model to predict SIC risk in patients with sepsis.

**Methods:**

Patients with sepsis admitted to the intensive care unit (ICU) between March 1, 2021, and March 1, 2024, at Hebei General Hospital and Handan Central Hospital (East District) were retrospectively included. Patients were categorized into SIC and non-SIC groups. Data were split into training (70%) and testing (30%) sets. Additionally, for temporal validation, patients with sepsis admitted between March 1, 2024, and October 31, 2024, at Hebei General Hospital were included. Feature selection was performed using least absolute shrinkage and selection operator (LASSO) regression and multivariate logistic regression. Nine ML algorithms were tested, and model performance was assessed using receiver operating characteristic curve (ROC) analysis, including area under the curve (AUC), calibration curves, and decision curve analysis (DCA). The SHaply Additive Explanations (SHAP) algorithm was used to interpret the best-performing model and visualize key predictors.

**Results:**

Among 847 patients with sepsis, 480 (56.7%) developed SIC. The random forest (RF) model with eight variables performed best, achieving AUCs of 0.782 [95% confidence interval (CI): 0.745, 0.818] in the training set, 0.750 (95% CI: 0.690, 0.809) in the testing set, and 0.784 (95% CI: 0.711, 0.857) in the validation set. Key predictors included activated partial thromboplastin time, lactate, oxygenation index, and total protein.

**Conclusions:**

This ML model reliably predicts SIC risk. SHAP enhances interpretability, supporting early, individualized interventions to improve outcomes in patients with sepsis.

## 1 Introduction

Sepsis is a life-threatening condition characterized by organ dysfunction organ resulting from dysregulated host response to infection, involving a dysregulated immune response and organ failure (1). Sepsis-induced coagulopathy (SIC) is a vascular endothelial cell injury and coagulopathy caused by sepsis (2). About 50% to 70% of patients with sepsis may develop coagulation disorders, and nearly 35% have secondary disseminated intravascular coagulation (DIC) (3, 4). Therefore, early identification of risk factors for coagulopathy in patients with sepsis to determine patients with SIC, and addressing the underlying causes of coagulopathy through appropriate prevention and treatment measures can significantly reduce mortality.

The concept of SIC was first proposed by the International Society for Thrombosis and Hemostasis (ISTH) in 2017, and it was previously used as one of the criteria for diagnosing sepsis (2). SIC is a complex pathophysiological state triggered by sepsis, characterized by a systemic inflammatory response with severe disturbances in the coagulation system (5). Its pathogenesis includes endothelial cell damage, release of inflammatory mediators, and excessive activation of coagulation factors, which may lead to DIC, multiple organ dysfunction syndrome, and even death (6–8). Inflammatory and coagulation responses have a synergistic effect: when the inflammatory response intensifies, the coagulation response is accelerated as a result (9). Therefore, when sepsis is exacerbated, it can lead to further activation of the coagulation system, causing systemic microvascular thrombosis and ultimately DIC, characterized by bleeding and microcirculatory failure (10, 11).

Machine learning (ML) is a branch of artificial intelligence that identifies patterns and relationships by analyzing a large amount of data (12). It has the advantages of self-learning, self-adaptation, high fault tolerance, and high efficiency by using algorithms to train models and analyze or predict new data using a trained model (13). In medical data analysis, ML algorithms are able to process complex high-dimensional datasets, identify potential disease risk factors, build predictive models, and assist physicians in diagnosis and treatment decisions (14). Recent studies have explored the role of ML in the prediction, diagnosis, and treatment of SIC and found that ML can enhance the early disease identification and diagnosis of SIC, facilitate personalized treatment of SIC, and improve the prognosis of patients with SIC (15).

The black-box feature of ML has limited its application in the medical field (16). The emergence of the SHaply Additive Explanations (SHAP) algorithm provides a new perspective to observe the predictive ability of ML models for different features, facilitating application and optimization (17). SHAP evaluates a feature’s impact on model predictions, highlighting the positive and negative effects of clinical variables on patient prognosis with enhanced explanatory power.

This study aimed to construct a clinical prediction model for early risk of patients with SIC using ML methods and to evaluate the optimal model using the SHAP algorithm. This approach seeks to enable early identification of patients who may develop SIC, assist clinicians in making clinical decisions faster, and facilitate timely interventions.

## 2 Methods

### 2.1 Data source

This retrospective study included patients with sepsis admitted to the intensive care unit (ICU) between March 1, 2021, and March 1, 2024, in the Hebei General Hospital and Handan Central Hospital (East District) with relatively complete data. Statistical software was used to randomly select 70% of the patient data as the training set to construct the prediction model, and then the remaining 30% of the patient data was used as the testing set. In addition, patients with sepsis admitted to ICU with relatively complete data from March 1, 2024, to October 31, 2024, in the Hebei General Hospital were retrospectively included as the temporal validation set. This study complied with the review and approval criteria of the Ethics Committee of Hebei General Hospital (No. 2024-LW-0223) and the Ethics Committee of Handan Central Hospital (No. 2024038).

### 2.2 Study subjects

Inclusion criteria: (1) Patients admitted to ICU for the first time; (2) Patients meeting the criteria for sepsis 3.0 as defined by the Society of Critical Care Medicine and European Society of Intensive Care Medicine in 2016.

**Figure 1:**
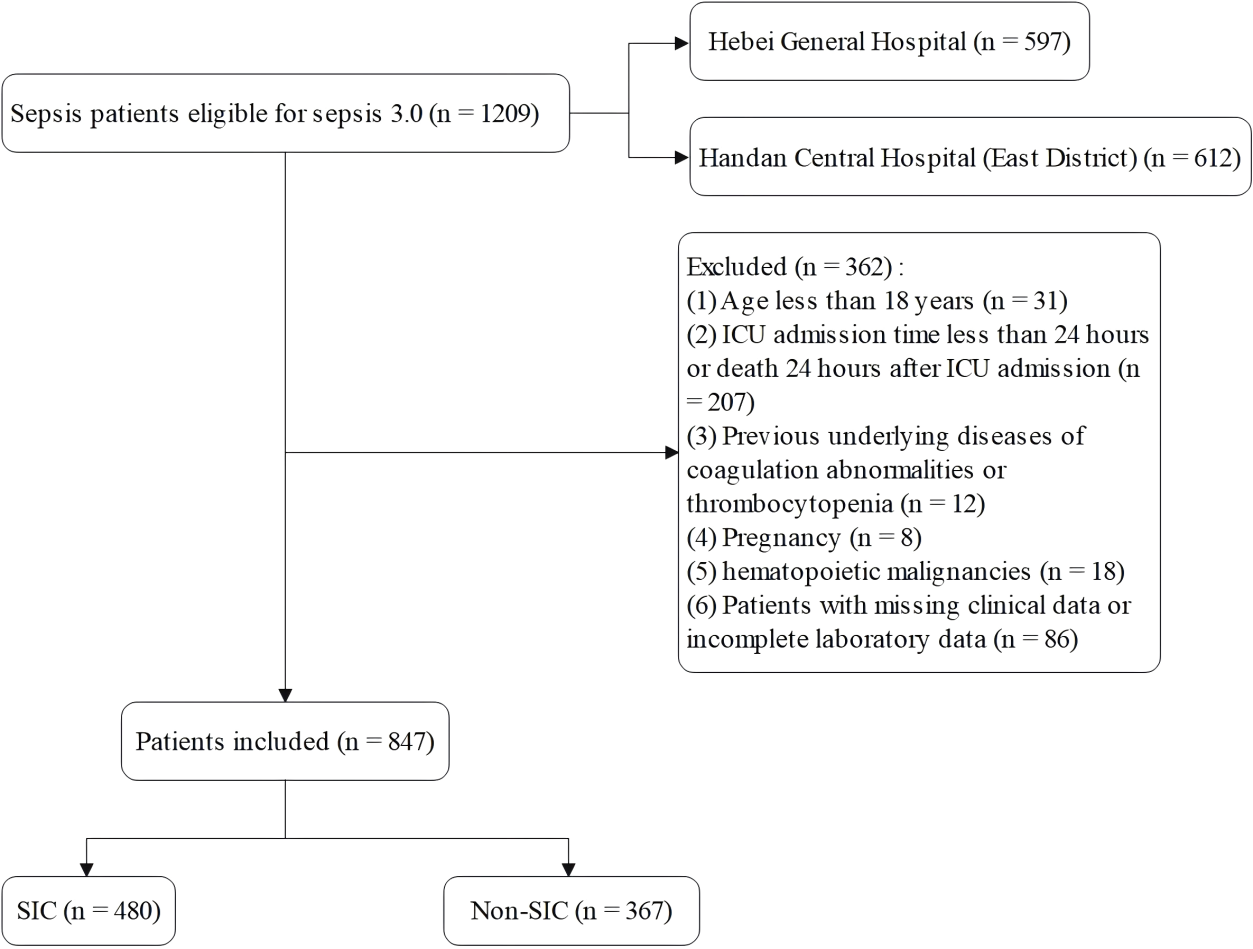
Patient screening flow.

Exclusion criteria: (1) Age less than 18 years; (2) ICU admission time less than 24 hours or death 24 hours after ICU admission; (3) Patients with pre-existing coagulation abnormalities or thrombocytopenia, such as thrombocytopenic purpura or hemophilia; (4) Patients with coagulation abnormalities before admission, such as pregnancy, hematopoietic malignancies, cardiopulmonary resuscitation, or sequential organ failure assessment (SOFA) scores less than 2; (5) Patients with missing clinical data or incomplete laboratory data. The patient screening process is shown in **Figure 1**.

Diagnostic criteria for SIC: The diagnostic criteria for SIC proposed by the ISTH 2017 were met. It was evaluated in terms of Prothrombin Time-International Normalization Ratio (PT-INR), platelet count, and all SOFA scores. PT-INR ≤ 1.2 was scored as 0 points, > 1.2 was scored as 1 point, and > 1.4 was scored as 2 points; platelet count ≥ 1.5×10^11^ was scored as 0, < 1.5×10^11^ was scored as 1, < 1.0×10^11^ was scored as 2; all SOFA scores were the sum of respiratory SOFA, cardiovascular SOFA, hepatic SOFA, and renal SOFA scores, which were scored as 0, 1, and 2, respectively, and when all SOFA scores were > 2, the score was still scored as 2. The diagnosis of SIC was made by the sum of the total scores of the above three aspects (PT-INR, platelet count, and all SOFA scores) of ≥ 4 and the sum of the PT-INR and platelet count scores of >2.

### 2.3 Data extraction

Data from the day before patients were diagnosed with SIC were extracted from electronic medical records at both hospitals. Variables extracted for this study were: (1) Demographic data: age and gender; (2) Underlying diseases: coronary atherosclerotic heart disease, hypertension, diabetes, chronic obstructive pulmonary disease, cerebrovascular disease, liver dysfunction, chronic kidney disease, malignancy, and history of surgery within 3 months; (3) Infection sites: pulmonary infection, abdominal infection, bloodstream infection, urinary system infection, central nervous system infection, skin and soft tissue infection, and other infections; (4) ICU disease severity scores: Acute Physiology and Chronic Health Evaluation II (APACHE II) and SOFA scores; (5) Vital signs: temperature, heart rate, systolic blood pressure, diastolic blood pressure, mean arterial pressure, and respiratory rate; (6) Laboratory tests: pH, lactic acid, actual bicarbonate (AB), oxygenation index (OI), base excess (BE), total protein (TP), albumin, total bilirubin (TBIL), direct bilirubin (DBIL), alanine aminotransferase (ALT), aspartate aminotransferase (AST), potassium (K), sodium (Na), chloride (Cl), calcium (Ca), phosphorus (PO_4_), magnesium (Mg), blood urea nitrogen (BUN), creatinine (Cr), procalcitonin (PCT), prothrombin time (PT), PT-INR, activated partial thromboplastin time (APTT), fibrinogen content, thrombin time, white blood cells (WBC), neutrophils, lymphocytes, monocytes, hemoglobin, red blood cell distribution width, platelet; (7) Interventions: deep venous catheterization, mechanical ventilation, anticoagulant drugs, hormones, vasoactive drugs, continuous renal replacement therapy, and infusion of human albumin.

### 2.4 Statistical analysis

The Shapiro-Wilk test was used to determine the normal distribution of the continuous variables. Normally distributed measurement data were expressed as mean (standard deviation), and the independent sample t-test was used for comparison between groups. Non-normally distributed measurement data were expressed as median (interquartile range), and the Wilcoxon rank sum test was used for comparisons between two independent groups. Enumeration data were presented as percentages (%), and the chi-squared test was used for group comparisons.

In order to exclude multicollinearity, variables with p-values less than 0.05 were included in the least absolute shrinkage and selection operator (LASSO) regression. Model complexity was penalized by applying a regularization term, setting uncorrelated or weakly correlated variable coefficients as 0. Large regression coefficients were selected to construct independent variables and dependent variable matrices. The LASSO regression model was stabilized using the lowest prediction error in 10-fold cross-validation (CV) to find the optimal λ value and determine the best predictor variables associated with the incidence of SIC in the training set. The final selected variables were included in multivariate logistic regression to identify independent risk factors for the development of SIC.

In this study, we constructed a clinical prediction model based on nine ML algorithms, including logistic regression (LR), naive bayes, support vector machine (SVM), extreme gradient enhancement (XGB), neural network (NN), random forest (RF), gradient elevator (GBM), K-nearest neighbor (KNN), and decision tree (DT). The selected independent risk factors were used to construct ML models to predict the development of SIC during hospitalization in patients with sepsis. In order to avoid model overfitting, a 10-fold CV was used for model training. Following successful model building, the performance of each model was assessed. The predictive value of each model was evaluated by plotting the receiver operating characteristic curve (ROC) and calculating the area under the curve (AUC), sensitivity, specificity, accuracy, F1 score, positive predictive value, and negative predictive value (NPV). The accuracy of the model was assessed by a calibration curve in which the horizontal coordinate is the predicted probability, the vertical coordinate is the actual occurrence probability. The ideal line is the diagonal line representing a perfect prediction, where the predicted probabilities exactly match the actual observed probabilities. The clinical utility of the prediction model was evaluated using a clinical decision curve analysis (DCA) curve. In the DCA plot, the None line represents an extreme case where the model predicts that all patients with sepsis will not develop SIC, indicating zero net clinical benefit. The All line represents the other extreme, where all patients with sepsis are predicted to develop SIC, at which point the slope of the net clinical benefit is negative. The net clinical benefit is determined by the range of threshold probabilities where the model’s curve lies above both the None and All lines. If the model curve is above the None and All lines, this suggests that the model has a higher net benefit in practical clinical applications. Finally, after a comprehensive evaluation of the AUC values, calibration curves, and DCA curves, the best predictive model was found, and the model was validated and evaluated again in the test set and validation set.

SHAP is based on the concept of Shapley values from cooperative game theory and uses an additive approach to compute the contribution of each feature to the predicted outcome. The SHAP method can provide an explanatory value for each feature that indicates the degree of influence of that feature on the predicted outcome of the model. It also provides a visualization tool for intuitively displaying the degree of influence of each feature on each data point, as well as for detecting interactions and non-linear relationships between features, further improving the explanatory and predictive power of the model.

In order to provide a global interpretation of the model, the SHAP algorithm generates a summary plot and a feature importance plot. In the summary plot, the x-axis (SHAP values) shows the magnitude of each feature’s impact on the predicted results, with points further away from the centerline (zero) indicating that the feature has a greater impact on the model’s output. The y-axis (feature ordering) ranks the features according to the magnitude of their impact on the target variable, from top to bottom. Pink dots indicate that the feature positively affects the target variable, while blue dots indicate that the feature has a negative impact on the target variable. The darker the color, the stronger the feature’s impact on the target variable.

The feature importance plot ranks features in order of their contribution to model predictions to provide insight into the relevance of each predictor variable. The top features with longer bars have a greater impact on the model predictions, while the bottom features with shorter bars have less influence.

For local model interpretation, the SHAP algorithm generates dependency graphs. These graphs show the impact of individual features on the predicted results of a ML model, with the x-axis representing the feature value and the y-axis representing the SHAP value (a measure of the importance of the feature on the predicted results). Each point in the dependency plot represents a patient. The position of each point indicates the SHAP value of a feature in that sample, with SHAP values greater than 0 indicating an increased risk in patients.

All statistical analyses were performed in SPSS (version: 27.0), R (version: 4.3.1), and Python (version: 3.12.7). P-values less than 0.05 were considered statistically significant. Data splitting was done using the initial_split function of the “rsample” package in R. Machine learning models, including logistic regression and random forests, were built using the “caret” package and cross-validated (10 times). Model calibration using the calibrate function of the “rms” package and DCA using the “dcurves” package.

## 3 Results

### 3.1 Baseline characteristics

A total of 847 patients with sepsis were screened, and 480 (56.7%) developed SIC. The patients were randomly divided into a training set and a testing set in a 7:3 ratio using R statistical software. The training set included 592, with 336 (56.8%) in the SIC, while the testing set comprised 255 patients, with 144 (56.5%) in the SIC group. The baseline data of patients in both sets are shown in **Supplementary Table S1**. Compared with the testing set, a larger proportion of patients in the training set had malignant tumors (*P* < 0.05). The rest of the indicators showed no statistical differences between the two data sets (*P* > 0.05), demonstrating that the data in the training and testing sets were well-balanced and comparable.

**Table 1** shows the baseline information for all patients with sepsis in the training set. A total of 592 patients with sepsis were included: 336 patients with SIC and 256 patients with non-SIC. Compared with patients with sepsis alone, patients with SIC had a greater proportion of underlying diseases, higher SOFA scores, and faster heart rate. Meanwhile, patients with SIC had higher concentrations of lactate, TBIL, DBIL, ALT, AST, Na, Cl, BUN, Cr, and PCT, as well as longer PT, APTT, and PT-INR, and a higher probability of deep venous cannulation. In contrast, patients with SIC have low pH, AB, OI, BE, TP, albumin, K, Ca, Mg, WBC, neutrophils, lymphocytes, monocytes, hemoglobin, platelet, and are less likely to be on anticoagulant medications. For other variables, no statistically significant differences were observed between the two groups (*P* > 0.05).

**Table 1:** Baseline comparison of SIC and non-SIC in training set.

Variables	SIC (n=336)	Non-SIC (n=256)	*P*
Demographic data			
Male, n(%)	210 (62.50)	166 (64.84)	0.557
Age, SD	70.01 (14.73)	71.01 (14.83)	0.415
Underlying diseases, n(%)			
Coronary atherosclerotic heart disease	71 (21.13)	56 (21.88)	0.827
Hypertension	142 (42.26)	119 (46.48)	0.305
Diabetes	87 (25.89)	72 (28.12)	0.544
Chronic obstructive pulmonary disease	37 (11.01)	32 (12.50)	0.576
Cerebrovascular disease	114 (33.93)	102 (39.84)	0.139
Liver dysfunction	151 (44.94)	84 (32.81)	0.003
Chronic kidney disease	91 (27.08)	57 (22.27)	0.18
Malignancy	36 (10.71)	37 (14.45)	0.17
History of surgery within 3 months	158 (47.02)	127 (49.61)	0.533
Infection sites, n(%)			
Pulmonary	220 (65.48)	178 (69.53)	0.298
Abdominal	142 (42.26)	101 (39.45)	0.491
Blood	44 (13.10)	33 (12.89)	0.942
Urinary system	53 (15.77)	55 (21.48)	0.075
Central nervous system	4 (1.19)	9 (3.52)	0.056
Skin and soft tissue	8 (2.38)	4 (1.56)	0.484
Other	17 (5.06)	7 (2.73)	0.155
ICU disease severity scores, M (Q_1_, Q_3_)			
APACHE score	24.00 [19.00, 29.00]	23.00 [18.00, 28.00]	0.063
SOFA score	9.00 [7.75, 12.00]	9.00 [7.00, 10.00]	<0.001
Vital signs, M (Q_1_, Q_3_)			
Temperature(°C)	36.50 [36.00, 37.20]	36.50 [36.00, 37.00]	0.137
HR(times/min)	100.00 [84.00, 113.00]	93.00 [82.00, 113.00]	0.047
SBP(mmHg)	121.00 [105.00, 140.00]	121.00 [105.00, 138.25]	0.997
DBP(mmHg)	66.00 [56.00, 76.00]	67.00 [57.00, 78.00]	0.308
MAP(mmHg)	85.00 [75.17, 94.67]	85.83 [75.92, 94.67]	0.601
RR(times/min)	21.00 [16.00, 28.00]	20.00 [16.00, 25.00]	0.281
Laboratory tests, M (Q_1_, Q_3_)			
pH	7.36 [7.29, 7.42]	7.39 [7.31, 7.44]	0.004
lactate(mmol/L)	2.68 [1.70, 4.70]	1.94 [1.40, 3.10]	<0.001
AB(mmol/L)	20.25 [16.50, 23.70]	22.00 [18.50, 25.52]	<0.001
OI	202.50 [135.12, 281.82]	245.20 [166.65, 354.35]	<0.001
BE(mmol/L)	-4.85 [-8.00, -1.50]	-2.88 [-6.30, 0.64]	<0.001
TP(g/L)	48.85 [42.90, 55.12]	53.90 [47.08, 61.28]	<0.001
ALB(g/L)(μmol/L)	26.70 [23.17, 30.92]	28.65 [24.98, 32.60]	<0.001
TBIL(μmol/L)	21.30 [12.50, 38.17]	14.40 [9.50, 21.83]	<0.001
DBIL(μmol/L)	9.55 [5.20, 20.45]	5.40 [3.20, 10.40]	<0.001
ALT(U/L)	32.10 [16.00, 78.93]	24.00 [13.00, 49.07]	0.001
AST(U/L)	61.50 [31.88, 134.73]	33.00 [21.00, 70.25]	<0.001
K(mmol/L)	4.00 [3.60, 4.50]	4.15 [3.70, 4.68]	0.038
Na(mmol/L)	140.40 [135.45, 145.00]	138.00 [135.00, 142.57]	0.014
Cl(mmol/L)	106.00 [101.60, 111.00]	104.00 [99.00, 108.28]	<0.001
Ca(mmol/L)	1.96 [1.83, 2.16]	2.02 [1.89, 2.16]	0.014
PO_4_(mmol/L)	1.14 [0.80, 1.51]	1.14 [0.82, 1.40]	0.72
Mg(mmol/L)	0.82 [0.70, 0.92]	0.86 [0.73, 0.99]	0.004
BUN(mmol/L)	13.69 [8.72, 21.33]	11.50 [7.24, 19.33]	0.01
Cr(μmol/L)	130.45 [81.80, 205.22]	98.90 [63.02, 158.32]	<0.001
PCT(ng/mL)	16.59 [2.90, 59.87]	3.90 [0.86, 26.38]	<0.001
PT(s)	16.10 [14.20, 18.10]	13.70 [12.40, 15.20]	<0.001
PT-INR	1.45 [1.31, 1.60]	1.16 [1.06, 1.30]	<0.001
APTT(s)	36.90 [32.70, 44.90]	32.30 [28.80, 36.62]	<0.001
FIB(g/L)	5.23 [3.42, 12.70]	5.91 [4.16, 11.92]	0.09
TT(s)	15.60 [5.63, 17.30]	15.40 [6.06, 17.20]	0.817
WBC(10^9^/L)	11.26 [5.95, 17.57]	12.95 [9.10, 18.56]	0.002
NEU(10^9^/L)	9.63 [4.98, 16.46]	11.46 [7.36, 16.64]	0.012
LYM(10^9^/L)	0.52 [0.32, 0.82]	0.80 [0.46, 1.30]	<0.001
MON(10^9^/L)	0.31 [0.14, 0.51]	0.47 [0.26, 0.84]	<0.001
Hb(g/L)	102.00 [88.00, 123.00]	109.50 [91.00, 128.00]	0.011
RDW(fL)	48.30 [45.00, 54.00]	48.70 [45.10, 52.85]	0.64
PLT(10^9^/L)	92.00 [52.00, 125.25]	232.50 [189.00, 299.50]	<0.001
Interventions, n(%)			
deep venous catheterization	279 (83.04)	190 (74.22)	0.009
anticoagulant drugs	147 (43.75)	137 (53.52)	0.018
mechanical ventilation	295 (87.80)	231 (90.23)	0.351
hormones	240 (71.43)	185 (72.27)	0.823
vasoactive drugs	319 (94.94)	247 (96.48)	0.364
CRRT	188 (55.95)	139 (54.30)	0.688
infusion of human albumin	256 (76.19)	177 (69.14)	0.055

Z, Mann-Whitney test; χ², Chi-square test.

M, Median; Q_1_, 1st Quartile; Q_3_, 3st Quartile.

SIC, sepsis-induced coagulopathy; APACHE, acute physiology and chronic health evaluation II; SOFA, sequential organ failure assessment; HR, heart rate; SBP, systolic blood pressure; DBP, diastolic blood pressure; MAP, mean arterial pressure; RR, respiratory rate; AB, actual bicarbonate; OI, oxygenation index; BE, base excess; TP, total protein; ALB, albumin; TBIL, total bilirubin; DBIL, direct bilirubin; ALT, alanine aminotransferase; AST, aspartate aminotransferase; K, potassium; Na, sodium; Cl, chloride; Ca, calcium; PO_4_, phosphorus; Mg, magnesium; BUN, blood urea nitrogen; Cr, creatinine; PCT, procalcitonin; PT, prothrombin time; PT-INR, prothrombin time-international normalisation ratio; APTT, activated partial thromboplastin time; FIB, fibrinogen; TT, thrombin time, WBC, white blood cells; NEU, neutrophils; LYM, lymphocytes; MON, monocytes; Hb, hemoglobin; RDW, red blood cell distribution width; PLT, Platelet.

### 3.2 Feature selection

**Figure 2:**
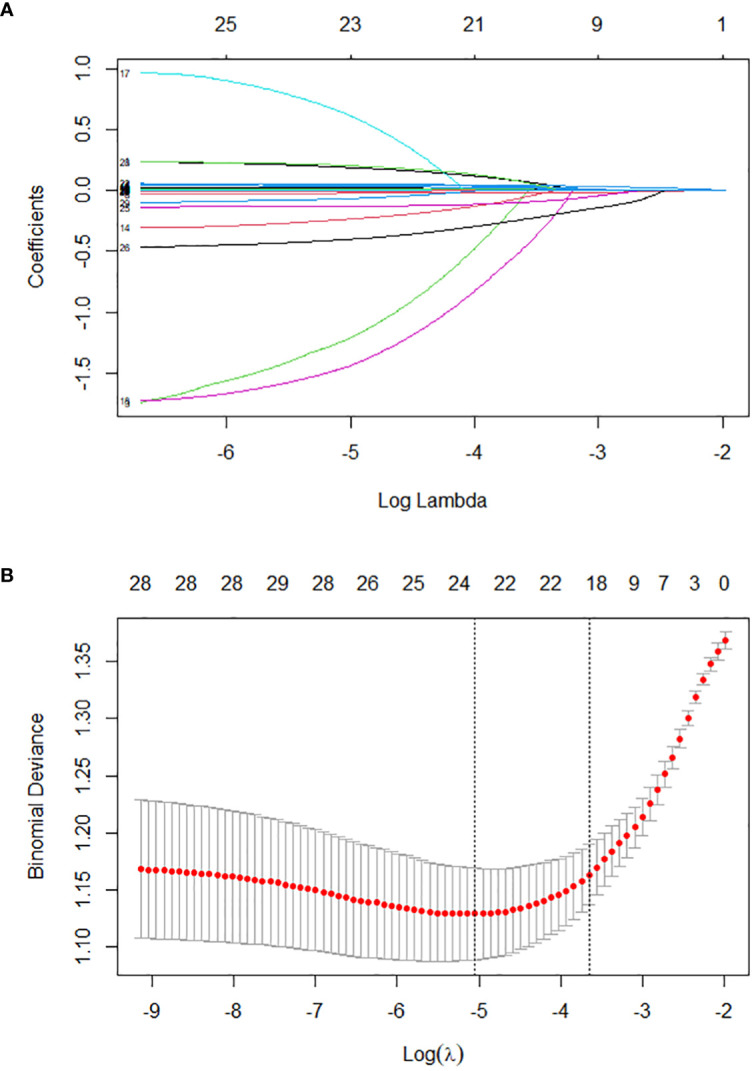
*Lasso regression-based variable screening.* **(A)**. Variation characteristics of variable coefficients; **(B)**. The process of selecting the optimal value of the parameter λ in the lasso regression model is carried out by the cross-validation method.

The training set was initially screened for relevant features using LASSO regression. LASSO regression included a total of 33 study variables with statistical significance in univariate LR. The regression model used the lowest prediction error in tenfold CV for screening analysis. LASSO regression was used to draw a dynamic process diagram of screening variables (**Figure 2A**) and a selection process diagram of cross-validated optimal parameter λ (**Figure 2B**). Finally, 19 variables were identified as being strongly associated with SIC: liver dysfunction, HR, pH, lactate, OI, TP, TBIL, AST, K, Cl, Mg, BUN, Cr, PCT, APTT, lymphocyte count, monocyte count, hemoglobin, and deep venous catheterization.

Significant predictive variables in LASSO regression were included in multivariate LR to identify independent risk factors for SIC. In order to construct a predictive model with good predictive performance and practical operability, the results showed that lactate, OI, TP, TBIL, BUN, PCT, APTT, and monocyte count were all independent risk factors for SIC. These variables were statistically significantly correlated with the occurrence of SIC (*P* < 0.05), as shown in **Table 2**.

**Table 2:** Multivariate logistic regression analysis of incidence related variables in patients with SIC.

Variables	β	SE	Wald	*P*	OR(95% CI)
lactate	0.078	0.039	4.072	0.044	1.081(1.002-1.167)
OI	-0.002	0.001	6.881	0.009	0.998(0.997-0.999)
TP	-0.032	0.01	10.757	0.001	0.969(0.951-0.987)
TBIL	0.017	0.004	15.548	<0.001	1.017(1.008-1.025)
BUN	0.021	0.008	6.916	0.009	1.021(1.005-1.036)
PCT	0.008	0.003	7.003	0.008	1.008(1.002-1.015)
APTT	0.061	0.011	29.657	<0.001	1.063(1.040-1.086)
MON	-0.628	0.195	10.338	0.001	0.534(0.364-0.783)
Constant	-0.702	0.673	1.089	0.297	

β, Partial regression coefficient; SE, Standard Error; OR, Odds Ratio; CI, Confidence Interval.

SIC, sepsis-induced coagulopathy; OI, oxygenation index; TP, total protein; TBIL, total bilirubin; BUN, blood urea nitrogen; PCT, procalcitonin; APTT, activated partial thromboplastin time; MON, monocytes.

### 3.3 Model performance comparisons

**Figure 3:**
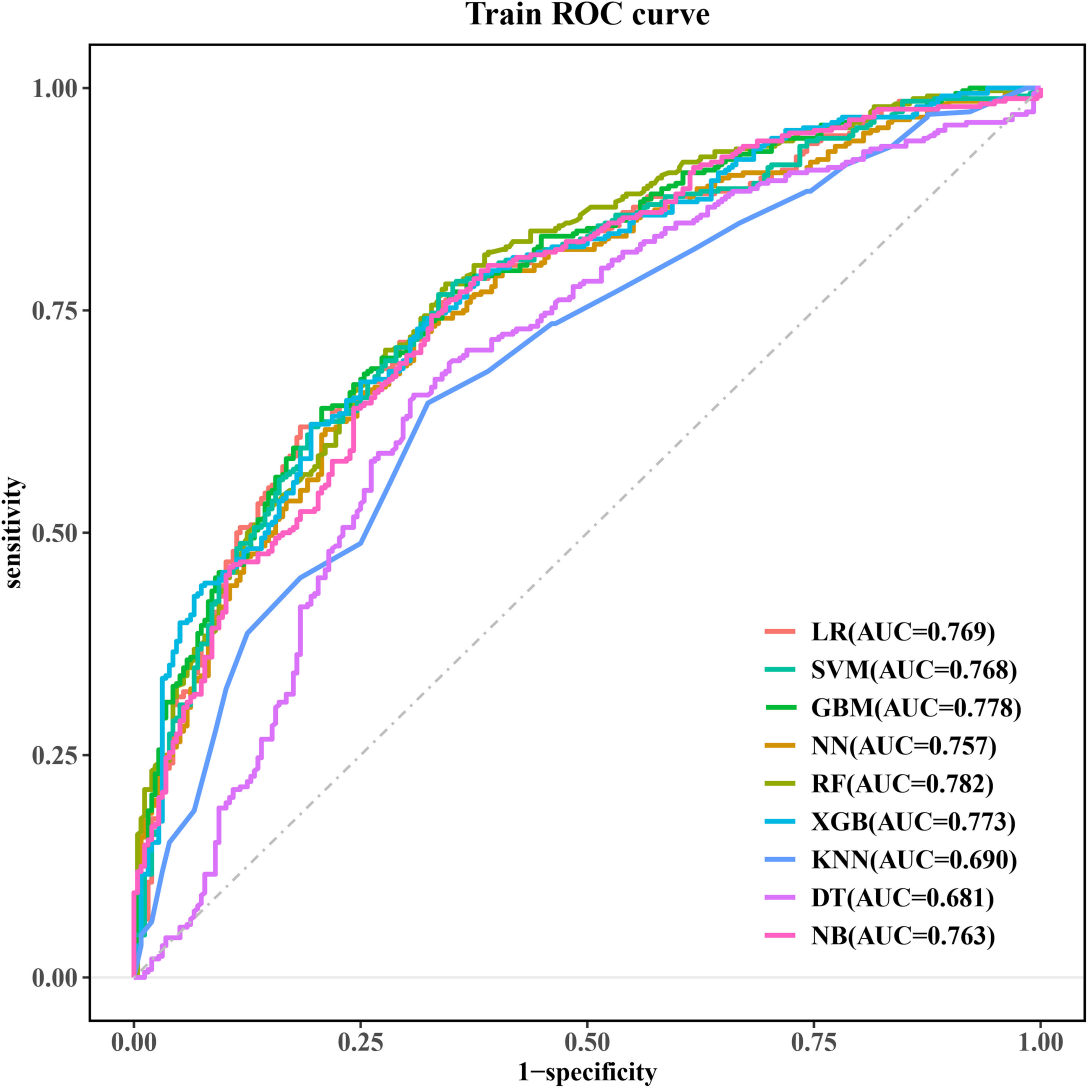
ROC curves for the machine learning models. ROC, receiver operating characteristic; LR, logistic regression; SVM, support vector machine; GBM, gradient elevator; NN, neural network; RF, random forest; XGB, extreme gradient enhancement; KNN, K-nearest neighbor; DT, decision tree; NB, naive bayes.

The eight independent risk factors (lactate, OI, TP, TBIL, BUN, PCT, APTT, and monocyte count) screened in multivariate LR were imported into nine ML algorithms to construct a model for predicting in-hospital occurrence of SIC in patients with sepsis. **Figure 3** shows the performance of the nine models in terms of ROC curves. All models showed good predictive performance for the onset of SIC, with the RF model performing best. The AUC value of the RF model was 0.782 [95% confidence interval (CI): 0.745, 0.818], followed closely by the GBM model with a comparable AUC value of 0.778 (95% CI: 0.741, 0.815). These models were superior to the other algorithms, although the other models still demonstrated good predictive ability. The remaining models were ranked in descending order of performance as follows: XGB (AUC = 0.773, 95% CI: 0.736, 0.810), LR (AUC = 0.769, 95% CI: 0.731, 0.807), SVM (AUC = 0.768, 95% CI: 0.730, 0.806), NB (AUC = 0.763, 95% CI: 0.724, 0.801), NN (AUC = 0.757, 95% CI: 0.718, 0.795), KNN (AUC = 0.690, 95% CI: 0.647, 0.732), and DT (AUC = 0.681, 95% CI: 0.637, 0.726).

**Table 3** shows the detailed performance metrics for the nine models. The RF model showed superior overall performance (sensitivity: 0.780, specificity: 0.656). Notably, RF achieved the highest F1 score (0.764) and accuracy (0.726) while possessing the highest NPV (0.694) among all assessed models.

**Table 3:** Performances of the machine learning models for predicting SIC.

Models	AUC	95% CI	Sensitivity	Specificity	PPV	NPV	Accuracy	F1 Score
LR	0.769	0.731-0.807	0.619	0.816	0.816	0.620	0.704	0.704
SVM	0.768	0.730-0.806	0.768	0.664	0.750	0.686	0.723	0.759
GBM	0.778	0.741-0.815	0.640	0.793	0.802	0.627	0.706	0.712
NN	0.757	0.718-0.795	0.723	0.688	0.752	0.654	0.708	0.738
RF	0.782	0.745-0.818	0.780	0.656	0.749	0.694	0.726	0.764
XGB	0.773	0.736-0.810	0.622	0.805	0.807	0.619	0.701	0.703
KNN	0.690	0.647-0.732	0.646	0.676	0.723	0.593	0.659	0.682
DT	0.681	0.637-0.726	0.655	0.691	0.736	0.604	0.671	0.693
NB	0.763	0.724-0.801	0.744	0.672	0.749	0.667	0.713	0.746

SIC, sepsis-induced coagulopathy; AUC, area under the curve; CI, confidence interval; PPV, positive predictive value; NPV, negative predictive value;

LR, logistic regression; SVM, support vector machine; GBM, gradient elevator; NN, neural network; RF, random forest; XGB, extreme gradient enhancement; KNN, K-nearest neighbor; DT, decision tree; NB, naive bayes.

**Figure 4:**
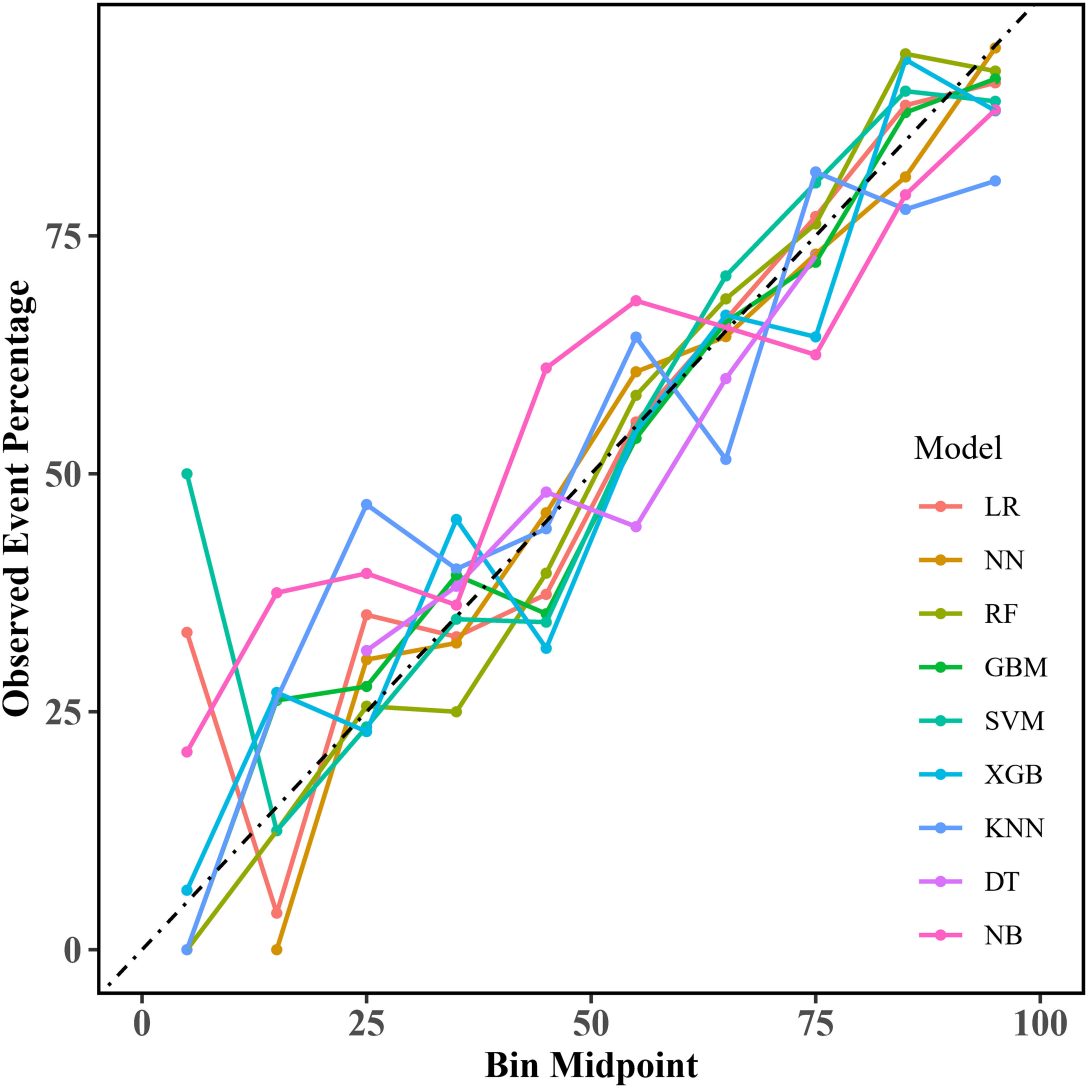
Calibration curves for the machine learning models. LR, logistic regression; NN, neural network; RF, random forest; GBM, gradient elevator; SVM, support vector machine; XGB, extreme gradient enhancement; KNN, K-nearest neighbor; DT, decision tree; NB, naive bayes.

Calibration curves for the nine models are shown in **Figure 4**, providing important insights into their predictive reliability. We also calculated the Brier score and calibration error to compare the degree of calibration of each model. The results show that the RF, GBM, and XGB models have lower Brier scores, with the RF model having the best prediction; in terms of calibration error, the NN, NB, and RF models have lower calibration errors, with the NN model having the most accurate prediction probability. The Brier scores and calibration errors of each model are shown in **Supplementary Table S3**. Thus, overall, all nine models showed better agreement between predicted probabilities and observations, but the RF model was better.

**Figure 5:**
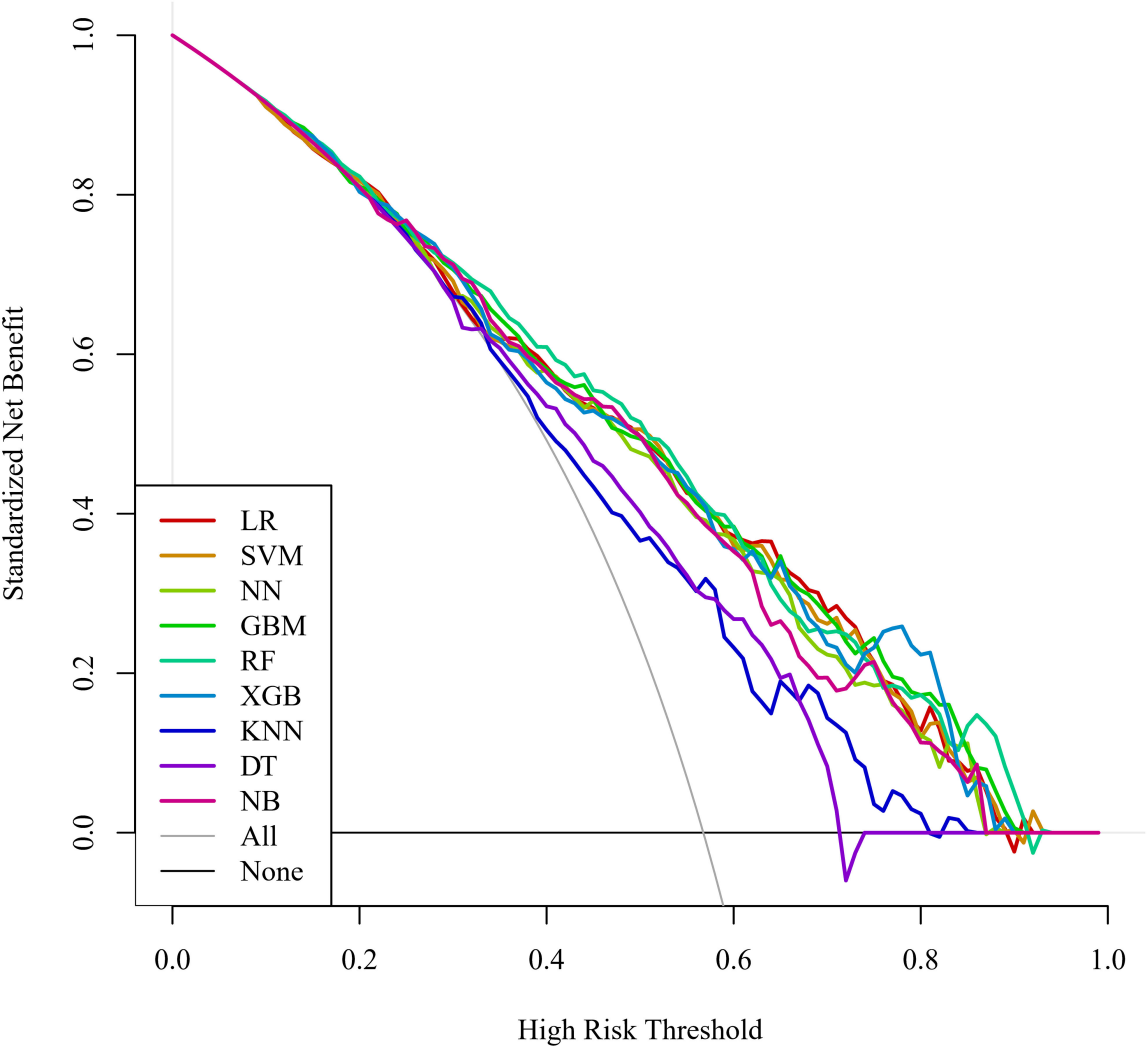
DCA curves for the machine learning models. DCA, decision curve analysis; LR, logistic regression; SVM, support vector machine; NN, neural network; GBM, gradient elevator; RF, random forest; XGB, extreme gradient enhancement; KNN, K-nearest neighbor; DT, decision tree; NB, naive bayes.

Regarding clinical utility, DCA curves were plotted in this study to assess the clinical utility of the model (**Figure 5**). Comparing the DCA curves, the RF model demonstrated the highest clinical utility in the high-risk range of 80% to 89% net benefit intervals. The use of the RF model to predict can increase more net benefits and is more clinically useful. In contrast, LR and XGB models showed better net benefit in the lower high-risk range (e.g., 0.6 to 0.8 or 0.7 to 0.9). After a comprehensive comparison of comparative AUC values, calibration curves, and DCA curves, the RF model was selected as the best model for predicting the occurrence of SIC.

### 3.4 Testing set

In the testing set, the LR, SVM, and RF models performed better. The LR model performed best, with an AUC value of 0.768 (95% CI: 0.710, 0.826). The SVM model followed with an AUC value of 0.765 (95% CI: 0.707, 0.823). The RF model ranked third with an AUC value of 0.750 (95% CI: 0.690, 0.809), which was superior to the remaining algorithms (**Supplementary Figure S1**).

### 3.5 Validation set

To validate the applicability of each model to a different dataset, this study systematically reviewed patients admitted to the Hebei General Hospital ICU with a diagnosis of sepsis from March 1, 2024, to October 31, 2024. The validation set flowchart is shown in **Supplementary Figure S2**, and the prediction items for the validation set are shown in **Supplementary Table S2**.

In the validation set, the XGB, RF, and SVM models performed better. The XGB model had the best performance, with AUC values of 0.785 (95% CI: 0.711, 0.858). The RF model closely followed with an AUC value of 0.784 (95% CI: 0.711, 0.857), which was superior to the remaining algorithms (**Supplementary Figure S3**).

### 3.6 Model visualization

**Figure 6:**
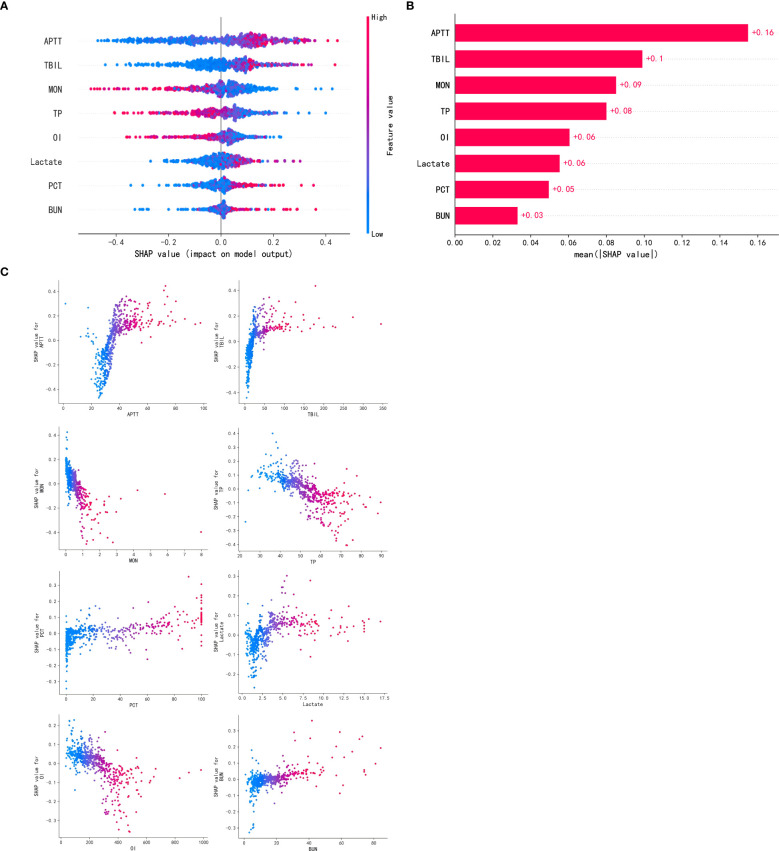
Visually interpret machine learning models using SHAP. **(A)**. SHAP summary plot. **(B)**. SHAP feature importance plot. **(C)**. SHAP dependency plots. APTT, activated partial thromboplastin time; TBIL, total bilirubin; MON, monocytes; TP, total protein; OI, oxygenation index; PCT, procalcitonin; BUN, blood urea nitrogen.

After comparing AUC values, calibration curves, and DCA curves across models, the RF model showed the best performance. Hence, we used the SHAP algorithm to analyze the interpretability of the RF model and SHAP plots were generated. The summary plot (**Figure 6A**) and feature importance plot (**Figure 6B**) show the (positive or negative) influence of features on the RF model. The top five factors influencing the risk of SIC in patients with sepsis were APTT, TBIL, monocyte count, TP, and OI. For instance, in the case of APTT, the coloring was deeper with increasing SHAP values, indicating that extreme values of this score can significantly increase the risk of developing SIC.

Additionally, a partial interpretation of the model was made, and the present study generated dependency plots for these eight clinical variables affecting the outcome (**Figure 6C**). The figure shows that longer APTT, higher TBIL, lower monocyte counts, lower TP, lower OI, higher blood lactate, higher procalcitonin, and higher BUN were associated with the risk of developing SIC in patients with sepsis. This graphical representation helps personnel better understand the specific contribution of each variable to the risk of developing SIC in patients with sepsis, thereby aiding in the prediction of disease progression and improving treatment options for patients.

## 4 Discussion

SIC is a vascular endothelial cell injury and coagulation disorder caused by sepsis (2). Globally, SIC occurs in 24.0% to 60.0% of patients with sepsis, while in China, the incidence is as high as 67.9%. Without proper management, SIC can develop into DIC, doubling morbidity and mortality rates (18, 19). The incidence of SIC in this study was about 56.7%, consistent with the global reported rate, although the figure remains considerably high. The ISTH and the European Society of Cardiology have issued diagnostic criteria and treatment guidelines for SIC. However, no diagnostic or therapeutic guidelines for SIC have been formulated in China so far (20, 21). There are also fewer studies related to SIC, which is related to the lack of attention to this disease by clinicians. Therefore, it is important to establish a prediction model for early identification and screening of patients with SIC.

With the rapid advancement of artificial intelligence, ML algorithms may reduce human assistance and improve classification accuracy (22). Compared to traditional models, ML can handle more complex, higher-dimensional datasets and achieve better accuracy than traditional models. However, many ML report AUC values for ROC curves when evaluating models, but AUC values do not fully reflect the predictive value of the model in clinical practice (23). Therefore, in addition to evaluating ML models, multiple metrics, such as calibration curves and DCA curves, should be integrated to comprehensively assess model performance and actual clinical utility value.

Zhao et al. used a dynamic prediction method using time series data modeling to predict the incidence of patients with SIC (15). They found that ML models predicted the incidence of patients with SIC more accurately than LR and SIC scores, with AUC values of 0.746 and 0.709 for LR and SIC scores, respectively. The Categorical Boosting (CatBoost) was the model with the highest AUC value among the ML models. The study relied heavily on continuous physiological and laboratory data and incorporated a missing value estimation strategy, highlighting the clinical utility of ML models for dynamic monitoring. In contrast, our RF model combined baseline and early-stage clinical characteristics, focused on key laboratory findings and vital signs on the day before SIC diagnosis, and did not require estimation due to rigorous data preprocessing. Additionally, our study used only eight variables, making the model relatively simpler. Furthermore, our study identified the optimal model by comprehensively evaluating metrics such as calibration curves and DCA curves and visualizing SHAP plots, demonstrating the efficacy of the RF model in the prediction of early-stage SICs and providing guidance for timely intervention.

Cui et al. (24) designed eight ML models to detect SIC and sepsis-associated DIC 8n (1 ≤ n ≤ 6) hours before SIC onset. The study developed an interpretable real-time sequential early warning model for real-world clinical data. The novel ODE-RNN model enabled continuous prediction at any time point, with predictions up to 8 hours in advance for SIC and DIC, achieving AUC values of 0.962 and 0.936 for SIC and DIC, respectively. They used a time-varying modeling approach, focused their study on clinical progression, and provided detailed SHAP-based feature significance. In contrast, our study focused on snapshot prediction using static data at an early stage, aiming for a simpler but effective approach. Although our study did not explicitly address disease progression, our results highlight the early predictive ability of RF models, which could complement the sequential modeling of Cui et al. Their study provides a sequential view of SIC progression, whereas our model prioritizes early risk stratification, providing a unique and synergistic perspective.

In this study, we used multi-center real-world clinical data to construct models using nine ML algorithms to predict the risk of SIC in patients with sepsis and applied the SHAP algorithm to perform an interpretable analysis of the optimal model. The results showed that the RF model exhibits good predictive performance with discriminatory and calibrated capabilities while providing substantial net benefits in clinical practice. Results from the validation set further confirmed the stability and accuracy of the model. In the RF model, APTT, TBIL, monocyte count, TP, and OI may be strongly associated with the risk of SIC in patients with sepsis.

The emergence of the SHAP algorithm provides a new perspective on the predictive ability of a model for different features and also facilitates the application and optimization of the model. The SHAP algorithm evaluates the significance of individual input features to the prediction of a given model and demonstrates the positive and negative impact of each clinical variable on the prognosis of a patient, with enhanced explanatory power. The present study generated predictive models that identify key risk factors but also made the models “interpretable,” successfully overcoming the black-box nature of ML. Good interpretability is essential not only for researchers to validate the reliability of new models but also for clinicians to gain confidence in sharing clinical decisions with ML models (25).

According to the SHAP algorithm, APTT was the most important predictive feature. In SIC, inflammatory mediators activate the coagulation system in a state of inflammation and coagulation disorders, and abnormal activation of endogenous coagulation pathways consumes large amounts of coagulation factors, resulting in prolonged APTT. Additionally, in sepsis, elevated levels of the thrombin-antithrombin complex indicate increased thrombin production, which further depletes coagulation factors and affects APTT (26). SIC is also accompanied by impaired anticoagulant mechanisms, such as decreased function of the protein C system and decreased antithrombin (27). Protein C insufficiency reduces factor Va and VIIIa inactivation and enhances coagulation, and APTT may be shortened or close to the lower limit but prolonged after factor depletion (28). In early SIC, the fibrinolytic system is transiently activated, increasing fibrinolytic enzymes to degrade fibrinogen and prolonging APTT. However, in later stages, fibrinolysis is inhibited, resulting in uncontrolled coagulation, with APTT affected by the complication (29). In summary, APTT is an important index for assessing coagulation function in patients with SIC. Prolonged APTT may suggest depletion of coagulation factors, activation of the fibrinolytic system, or impaired anticoagulation mechanisms. For therapeutic decision-making, the results of APTT can help physicians determine whether coagulation factor supplementation, anticoagulation therapy, or regulation of the fibrinolytic system is needed.

In SIC, inflammation involves the liver, and systemic inflammatory response syndrome causes hepatic dysfunction, which affects bilirubin uptake, binding, and excretion, leading to elevated TBIL levels (30). Impaired liver function also reduces coagulation factor synthesis, leading to coagulation abnormalities. In sepsis, oxygen free radicals are generated to induce oxidative stress, and bilirubin scavenges free radicals. Elevated levels may be a compensatory response, but excessively high levels reflect severe inflammation and oxidative stress, which damage endothelial cells, activate the coagulation system, and initiate the endogenous coagulation pathway (31). SIC triggers microcirculatory disturbances, which are manifested by thrombosis and inadequate perfusion. Changes in bilirubin levels may be associated with alterations in the microcirculation, which can further exacerbate hepatic injury and metabolic disturbances, resulting in malignant coagulation and metabolic disorders, forming a vicious circle (32). Monitoring TBIL levels in patients with sepsis is helpful in assessing the severity and prognosis of the disease in clinical practice. Persistent elevation of TBIL may signal ongoing liver dysfunction, worsening coagulation dysfunction, and an increased risk of multi-organ failure. From a treatment perspective, improving liver function and reducing inflammatory response and oxidative stress are important aspects of treating SIC. Monitoring changes in TBIL levels can provide a reference basis for adjusting therapeutic regimens.

Monocytes are a type of leukocyte, generated from bone marrow hematopoietic stem cells, which can differentiate into macrophages. Their functions include phagocytosis of pathogens, antigen presentation and secretion of cytokines, participation in sepsis-related immune responses, and regulation of inflammation (33). In SIC, monocyte counts may decrease due to myelosuppression caused by inflammatory mediators that inhibit bone marrow hematopoiesis (34). Decreased monocytes impair pathogen phagocytosis and inflammatory mediator secretion, leading to the spread of infection, which sustains activation of the coagulation system, depletes coagulation factors, and triggers coagulation disorders (35). Additionally, monocytes regulate T and B cell function, and their reduction triggers an immune imbalance that makes it difficult to clear pathogens and terminate excessive inflammation, exacerbating coagulation disorders. Hence, decreased monocyte count is usually a sign of severe disease. In patients with SIC, a persistent decrease in monocyte count often signals a poor prognosis, as it may indicate severe bone marrow suppression and a persistent decline in the body’s immune function and ability to regulate the inflammatory response. This increases the patient’s risk of multi-organ failure and makes it more challenging to correct coagulation dysfunction, as restoration of normal inflammatory response and immune function is one of the most important prerequisites for improving coagulation status.

Coagulation factors (e.g., II, VII, IX, X) are proteins synthesized by the liver. Liver dysfunction caused by sepsis leads to reductions in TP and coagulation factors, promoting the development of SIC. Hypoproteinemia reduces colloid osmotic pressure, leading to blood concentration, activation of coagulation, and weak resistance to inflammation, further aggravating coagulation disorders (36). The protein C system, which regulates coagulation, is dependent on TP levels, and its abnormalities can disrupt anticoagulant homeostasis, leading to uncontrolled coagulation (37). Thus, a decrease in TP level could indicate poor prognosis in patients with sepsis, as it is associated with a variety of adverse conditions such as coagulation dysfunction and uncontrolled inflammatory response. Correcting hypoproteinemia may help improve coagulation status, reduce inflammatory response, and improve survival.

In patients with sepsis, acute lung injury or acute respiratory distress syndrome can result from systemic inflammatory response. This inflammatory response increases pulmonary capillary permeability, promotes alveolar exudation, and decreases lung compliance, leading to impaired gas exchange and decreased OI. In sepsis, endotoxins and inflammatory mediators can activate the coagulation system, leading to extensive microthrombosis within the microcirculation (38). These microthrombi affect blood perfusion to vital organs such as the lungs, further exacerbating lung injury and decreasing the OI. The inflammatory response can also cause vascular endothelial cell damage, promote platelet aggregation and coagulation factor activation, aggravate coagulation dysfunction, and affect the gas exchange function of the lungs, which decreases the OI (39). Additionally, patients with SIC often suffer from multiple organ dysfunction, with impaired lung function being particularly common. Severe coagulation dysfunction can lead to complications such as pulmonary hemorrhage and pulmonary infarction, further reducing the OI. Thus, the OI is closely related to SIC, with the two conditions influencing each other and collectively reflecting the severity of patients with sepsis. Clinically, monitoring the OI helps to assess the pulmonary functional status and prognosis of patients with SIC.

Hyperlactatemia is closely associated with the development of patients with SIC, with two primary mechanisms underlying this association. First, elevated lactate damages endothelial cells and alters endothelial cell permeability, which in turn initiates exogenous coagulation and leads to coagulation dysfunction (40, 41). Second, elevated lactate can also cause acidosis, which has been found to inhibit thrombin generation. This coagulation dysfunction may result in the formation of microthrombi, which further aggravates peripheral circulatory hypoperfusion. Worsening hypoperfusion promotes lactate elevation, creating a vicious cycle of metabolism and coagulation that adversely affects patients (42, 43). Therefore, monitoring the change of lactate in patients with sepsis is critical for early identification of SIC, determining the stage of coagulation, understanding the degree of tissue hypoperfusion and organ hypoxia, and guiding interventions such as fluid resuscitation to maintain the homeostasis of the internal environment, prevent SIC, and improve prognosis.

In SIC, elevated PCT stimulates the release of von Willebrand factor from the vascular endothelium, which promotes microthrombosis and exacerbates coagulation disorders (44). Elevated PCT is indicative of an infectious or inflammatory stress state, which activates the coagulation and fibrinolytic systems. Activation of the coagulation system predisposes the blood to coagulation and microthrombosis while inhibiting early activation of the fibrinolytic system, further exacerbating coagulation abnormalities (45). Coagulation factors such as V and VIII are activated and depleted, leading to abnormal function. Studies have shown that higher levels of PCT in patients with SIC are associated with higher morbidity and mortality rates, and the level of PCT in patients with SIC is higher than that in patients with sepsis without coagulation disorders (46, 47). In patients with SIC, the combination of PCT levels and coagulation tests allows for a more comprehensive assessment of the disease. Adjusting the anti-infective regimen and coagulation modification therapy (e.g., anticoagulation or coagulation factor supplementation) according to changes in PCT is a crucial clinical strategy to improve the prognosis of patients.

SIC is often accompanied by renal impairment, as sepsis causes inadequate renal perfusion and decreased filtration rate, leading to BUN accumulation and elevated BUN (48). Renal injury also reduces the clearance of coagulation factors and fibrin degradation products and decreases erythropoietin levels, causing anemia and increased blood viscosity, which exacerbate coagulopathy (49, 50). Sepsis causes hypercatabolism, increases BUN production, and affects coagulation factor synthesis. The liver may reduce coagulation factor synthesis due to metabolic disturbances, leading to coagulopathy (51). Inflammatory mediators are released in sepsis, constricting the renal vasculature and decreasing BUN excretion while activating the coagulation system and initiating exogenous coagulation pathways. The interaction between elevated BUN and coagulation abnormalities aggravates the condition (38). Hence, monitoring BUN levels is important for assessing renal function and disease progression in patients with SIC. Persistently elevated BUN levels may indicate continued deterioration of renal function and may also signal worsening of coagulation dysfunction. By monitoring BUN levels, physicians can make timely adjustments to the treatment plan, such as adjusting the strategy of fluid resuscitation to improve renal perfusion and control the inflammatory response.

This study has several advantages. It used an innovative approach to build an interpretable ML prediction model for SIC risk in patients with sepsis. The model was developed from multi-center clinical data and validated to ensure it is representative. Additionally, the SHAP algorithm enhanced the model’s interpretability. The RF model showed good generalizability in the validation set, and the selected variables were common clinical indicators, making it easy to promote the model.

This study also has some limitations. First, this study is a retrospective study, which may be subject to selection bias and information bias, and future large-scale prospective studies are needed to further determine the robustness and value of the predictors and analyze the potential predictors to improve the inferential power of the results. Second, although this was a multi-center study and was internally and externally validated, the overall sample size was less than 2,000 cases from two hospitals in China with the same level of medical care. Patients with sepsis from other countries and different healthcare institutions were not included or discussed in this study. Hence, the applicability and generalizability of the model to different populations and settings may be limited. Future studies may consider using data from international public databases and multiple healthcare organizations to evaluate the performance of these models in a wider range of settings. In addition, although the time period validation used in this paper is one of the types of external validation, in practice external validation usually involves evaluating models in completely different environments. Therefore, the current validation method has some limitations, and there is a strong need for true external validation in future work. For the treatment of missing values, the proportion of assessed missing values was so small (< 5%) that it was unlikely to cause significant bias, and thus cases with missing values were excluded to maintain the integrity of the analysis. However, we recognize that the exclusion of missing values may introduce bias, especially in cases where the missing data mechanisms were missing at random (MAR) and missing not at random (MNAR). In future studies, we will explore advanced techniques such as inverse probability weighting or Bayesian methods for MNAR data and further validate our findings. Finally, all the data in this study were collected manually, and some missing clinical variables could not be analyzed during the collection process due to the differences in the medical conditions in each hospital, such as various culture results. Therefore, additional data support and in-depth analysis are needed. Despite these limitations, we believe that the RF model can help early identification of patients with SIC by clinicians.

## 5 Conclusion

We developed an ML model to predict the risk of SIC in patients with sepsis and validated its potential as a clinically reliable tool. The SHAP algorithm can improve the interpretability of ML model, help clinicians to better understand and apply the model, identify the main risk factors for SIC, and assist clinicians in implementing individualized and precise interventions at an early stage to prevent and reduce poor prognosis in patients with sepsis.

## Data Availability

The original contributions presented in the study are included in the article/**Supplementary Material**. Further inquiries can be directed to the corresponding author.
